# The Effect of Emotion Regulation Strategies on Functioning in Individuals with Bipolar Disorder

**DOI:** 10.1192/j.eurpsy.2025.1092

**Published:** 2025-08-26

**Authors:** R. Ucak, C. H. Ayhan, M. C. Aktas, S. Aktas

**Affiliations:** 1 Psychiatric Nursing; 2 Psychiatri, Van Yuzuncu Yıl University, Van, Türkiye

## Abstract

**Introduction:**

Bipolar disorder is a chronic disorder characterized by intense fluctuations in mood, which seriously affects the mental health and quality of life of the individual (APA, 2022). The most prominent features of this disorder include episodes of mania, hypomania and depression. In between these episodes, individuals may generally experience periods of emotional stability; however, the severity and frequency of emotional fluctuations may show individual differences. Bipolar disorder affects not only individuals’ mood, but also their cognitive functioning, social relationships, and overall mental health. In this context, the strategies these individuals use to cope with emotional difficulties play an important role in mental health. Especially in manic episodes, individuals often struggle with an uncontrolled increase in excessive positive emotions, while in depressive episodes they have to cope with intense negative emotions (Gruber, 2011). Research shows that individuals with bipolar disorder often resort to dysfunctional emotion regulation strategies, which may have a negative impact on the frequency and severity of episodes. However, studies on the effectiveness of emotion regulation strategies in individuals with bipolar disorder are limited. In particular, more research is needed on how these strategies play a role in different stages of the illness and to what extent the effects of these strategies on individuals’ overall functioning differ.

This study aims to examine the effects of emotion regulation strategies used by individuals with bipolar disorder on functioning.

**Objectives:**

The aim of this study is to examine the effect of emotion regulation strategies used by individuals with bipolar disorder on their functioning. In particular, it is aimed to analyze the differences between functional and non-functional strategies on functioning.

**Methods:**

This study was planned according to a descriptive correlational design. Data will be collected from individuals diagnosed with bipolar disorder using the Cognitive Emotion Regulation Scale, Difficulty in Emotion Regulation Scale-Short Form (DERS-16), Young Mania Rating Scale, Brief Psychiatric Functioning Rating Scale and Sociodemographic Data Form. Participants will fill in these scales and the effect of emotion regulation strategies on functioning will be evaluated. The data will be subjected to appropriate methods for statistical analysis.

**Results:**

Data extraction is still on going in detailed style by principal authors. Description of studies and the key findings will be presented.

**Conclusions:**

The results of this study aim to determine the different effects of functional and dysfunctional strategies on functioning in individuals struggling with bipolar disorder and to provide recommendations for interventions that can improve the mental health of these individuals.

**Key Words:**

bipolar disorder, emotion regulation, functioning, coping.

**Disclosure of Interest:**

None Declared

